# HIV and hepatitis C virus infections among hanka injection drug users in central Ukraine: a cross-sectional survey

**DOI:** 10.1186/1477-7517-6-23

**Published:** 2009-08-23

**Authors:** Kostyantyn V Dumchev, Ruslan Soldyshev, Han-Zhu Qian, Olexandr O Zezyulin, Susan D Chandler, Pavel Slobodyanyuk, Larisa Moroz, Joseph E Schumacher

**Affiliations:** 1 Vinnitsya Regional Narcological Dispensary, Vinnitsya, Ukraine; 2 University Hospital, Columbia, Missouri, USA; 3 Department of Health Services Research, Vanderbilt University, Nashville, Tennessee, USA; 4 Division of Preventive Medicine, Department of Medicine, The University of Alabama at Birmingham, Birmingham, Alabama, USA; 5 Vinnitsya National Medical University – Pirogov, Vinnitsya, Ukraine

## Abstract

**Background:**

Ukraine has experienced an increase in injection drug use since the 1990s. An increase in HIV and hepatitis C virus infections has followed, but not measures of prevalence and risk factors. The purposes of this study are to estimate the prevalence of HIV, HCV, and co-infection among injection drug users (IDUs) in central Ukraine and to describe risk factors for HIV and HCV.

**Methods:**

A sample of 315 IDUs was recruited using snowball sampling for a structured risk interview and HIV/HCV testing (81.9% male, 42% single, average age 28.9 years [range = 18 to 55]).

**Results:**

HIV and HCV antibodies were detected in 14.0% and 73.0%, respectively, and 12.1% were seropositive for both infections. The most commonly used drug was hanka, home-made from poppy straw and often mixed with other substances including dimedrol, diazepines, and hypnotics. The average period of injecting was 8.5 years; 62.5% reported past-year sharing needles or injection equipment, and 8.0% shared with a known HIV-positive person. More than half (51.1%) reported multiple sexual partners, 12.9% buying or selling sex, and 10.5% exchanging sex and drugs in the past year. Those who shared with HIV positive partners were 3.4 times more likely to be HIV positive than those who did not. Those who front- or back-loaded were 4 times more likely to be HCV positive than those who did not.

**Conclusion:**

Harm reduction, addiction treatment and HIV prevention programs should address risk factors to stop further spread of both HIV and HCV among IDUs and to the general population in central Ukraine.

## Background

Ukraine is the second largest country in Eastern Europe, with an estimated population of about 48 million. Political independence in Ukraine and surrounding Eastern European countries in the early 1990s has been associated with a rapid increase in the supply, use, and negative public health consequences of illicit drugs [1]. The number of persons officially registered at psychiatric/narcological facilities as drug-dependent patients increased from 51,484 in 1996 to 80,145 in 2008 [2]. The increased prevalence of narcotics addiction and demand for opiate drugs, along with the high cost of imported heroin in a developing country, have prompted expansion of cheaper intravenous solutions that are home-made from locally grown poppy. Home-made acetylated opioid solution, called hanka, is currently the most commonly used intravenous addictive drug in Ukraine, as opposed to Russia and Belarus, where heroin is more easily available[3].

The main source of hanka is locally grown poppy, which can be harvested for latex during June and July or dried for later use. Poppy latex can be used in a number of ways. It can be dissolved and injected; dried and smoked as opium; dried on a glass, scratched off and then snorted; and ingested. Poppy seed capsules and stems can be collected, dried and crushed into poppy straw for later use. Straw can be chewed and ingested or brewed in water to make poppy tea (kuknar), but the most common processing method is to refine the straw into acetylated morphine by a simple acid-base extraction. This process, involving solvents and acetic anhydride, produces a dark solution known as hanka or shirka that can be injected intravenously or intramuscularly. Users reported switching to intramuscular injection when peripheral veins obliterated (burned out) and phlebitis or personal discomfort prevented use of central veins.

The reported number of cases of HIV in Ukraine increased from 398 at the beginning of 1995 to 141,277 at the beginning of 2009 [4]. The true number of HIV cases among adults aged 15–49 by the end of 2007 was estimated to be somewhere between 230,000 and 573,000 [5], which yields an overall prevalence of 1.63%. The few available local sources show that HIV disproportionately affects injection drug users (IDUs), and regional prevalence ranges from 8.7% to 58.3% [6-8]. Hepatitis C virus (HCV) is even more common in IDUs, with regional prevalence ranging from 62% to 88% [7,9,10]. While HIV may be transmitted through multiple routes, HCV in IDUs is transmitted mainly via percutaneous exposure. A recent study showed that positive HIV status among Ukrainian IDUs was associated with female sex, daily injections, combined stimulant/opiate use, and having sex with HIV positive persons [11]. Thus, the risk factors for HIV and HCV infections among IDUs may differ and therefore lead to the different prevalence for the two infections. The purpose of this study was to document: 1) prevalence of HIV, HCV, and co-infection; 2) the current nature and extent of non-heroin opiate IDU patterns; and 3) sex- and drug-related risk factors that may differentiate HIV and HCV infections in an understudied rural area of central Ukraine.

## Methods

### Study setting

Vinnitsya is a mid-size oblast (region) in central Ukraine with a total population of 380,000, and the majority of local residents are farmers. At the end of 2008, a total of 1,299 HIV/AIDS cases have been registered in Vinnitsya Oblast. Most of these infections were attributed to IDU, although the proportion of IDUs among newly registered cases has declined from 77% in 1998 to 31.5% in 2008 [12]. The official number of persons in the Vinnitsya Oblast registered as drug-dependent patients at the Regional Narcological Dispensary (RND) at the end of 2008 was 1,146 [13]. However, the estimated figure is 5 to 7 times greater due to under-reporting [14]. This cross-sectional survey was conducted in 2005 to investigate the prevalence of HIV and HCV and associated risk factors among opiate IDUs in Vinnitsya, Ukraine, in collaboration with local investigators from the Infectious Disease Hospital, Vinnitsya National Medical University – Pirogov and the RND. The Fogarty International Center of the National Institutes of Health funded the study.

### Study participants

Study participants were recruited using a snowball sampling technique [15]. Seven initial "seeds" were recruited through the registered patient population of the RND. They were screened for the history of active injection drug use and also were selected to represent different city districts in the Vinnitsya Oblast. Upon discharge, they were offered the opportunity to refer their drug injecting peers for HIV testing and counseling for a referral fee ($2 US per referral). There was no limit on the number of referrals by each seed. This approach worked very well; within several weeks, the number of eligible IDUs willing to participate almost exceeded the testing capacity of the study site. The desired sample size was reached in three months. We estimate that our sample represented 15 to 20% of the IDU population in the city, based on the official registry of drug-dependent persons maintained by the RND.

Inclusion criteria for participating in the study included: 1) Age ≥ 18; 2) self-reported history of at least three injections of illicit drugs in the past 30 days; 3) residence in Vinnitsya for the past year; and 4) ability and willingness to give informed consent. The self-reported history of illicit drug injection was confirmed by probing knowledge of drug-related issues or the observed presence of physical needle marks. This criterion was not used to rule out new or careful users, but rather non-users attempting to earn the study incentive. No volunteers were excluded for lack of physical stigmata alone, nor was prior HIV or HCV testing an exclusion criterion. The study was approved by the institutional review and scientific ethics boards of The University of Alabama at Birmingham and the Vinnitsya National Medical University – Pirogov.

### Data collection

After the initial eligibility screening, research staff (KD, RS, OZ) explained the study to each participant; obtained written informed consent; and provided participants with HIV pre-test counseling, following the US Centers for Disease for Control (CDC) guidelines [16] and complemented by a corresponding HCV protocol created by the investigators. A structured interview was then administered by trained interviewers at the Infectious Diseases Hospital. It included demographic information, HIV knowledge (HIV-KQ-45), the Form 90 for lifetime and past 30-day drug use history, and the Risk Assessment Battery (RAB) for sex- and drug-related HIV risk behaviors in the past 6 months [17-19]. Participants also were asked if they ever used needle exchange programs, which were available in the study locale during the time that this research was conducted. Qualitative information on culturally specific aspects of drug purchase, use and preparation also was collected in a structured interview. Research staff asked participants to describe the nature of injection practices, types of drugs used, purchasing practices, opiate preparation practices, and modes of IDU. Finger prick whole blood samples were collected to test for HIV and HCV antibodies using ACON rapid HIV and HCV tests (ACON Laboratories, GBI Biotech Co., Ltd., Beijing, China) after pre-test counseling. Sensitivity and specificity are reported to reach 99% per the test manual. All participants then were provided with post-test counseling for both HIV and HCV results, per CDC guidelines and study protocol, before they left the study clinic. Participants with reactive HIV and/or HCV screening tests were referred to local facilities for diagnostic confirmation and other medical services. Participants were compensated for their time and effort with monetary vouchers worth 30 Grivna (about 6 US dollars).

### Data analysis

Statistical analyses were performed using SAS version 9.1 (SAS Institute Inc., Cary, NC, USA). Baseline point prevalence was calculated for HIV and HCV seropositivity. Univariate analyses were undertaken first to identify factors associated with HIV and HCV infections, which included demographic variables, sex and drug use risk behaviors, and harm reduction experience. Variables significantly associated with infection status in univariate models (*P *< 0.05) were included in multivariate logistic regression models. Those variables that were not significant in the multivariable models at the significance level of 0.05 were eliminated in a stage-wise manner, identifying variables that were independently associated with HCV and HIV seropositivity. Observations with missing values in any variables included in the models were excluded from analysis. Use of recent behaviors as predictors of prevalent infections is based on the assumption that the behaviors did not vary post-infection. That is a limitation to this analytic approach.

## Results

### Demographics of participants

Among the 380 persons who self-reported injection of illicit drugs within the past 30 days, 4 were younger than age 18. Another 61 were ineligible due to the absence of physical drug injecting signs in combination with poor knowledge of the local IDU scene and practices and/or unconvincing statements about personal drug use. Thus, 315 participants were enrolled in this study. Of these, 81.9% were males; 42.0% were single; average age was 28.9 years (range 18–55); 38.1% had at least some college (post-secondary) education; 31.7% were unemployed; and 1.3% were homeless.

### Drug abuse characteristics

In this study, more than ninety percent (93.6%) reported intravenously injecting hanka in the past month, and 1.3% were injecting it intramuscularly; 9.3% ingested opiates (through chewing or brewing poppy straw); 7.6% smoked opium; and 4.2% snorted dried poppy juice. Hanka is made either by users themselves or by a dealer, who may be a user as well. In both cases, the solution often is drawn up from a common container; 70% of the participants reported using this practice in the past year. The solution also may be back- or front-loaded from another syringe (72% in the past year). Dealers usually use a large 20 ml syringe with a bent needle to front-load buyers' smaller syringes, which creates a possibility for mass contamination if one of the buyers uses an infected syringe.

Different substances often were added to the hanka solution before injecting in order to boost or prolong its desired effects, minimize adverse effects (e.g. nausea and hyperthermia), and improve chemical properties (e.g., making the solution look more clear). Participants reported that whole blood was used in the past to precipitate debris during preparation of these drugs, but adding other substances to alter the subjective effect has become more common. Only 14.9% of participants reported using pure hanka over the past month. The majority mixed it with dimedrol (antihistamine medication, 77.2%), tranquilizers (benzodiazepines, 15.4%), or hypnotics (diazepines and others, 2.5%). Substances used concurrently with opiates (or shortly thereafter) included an injectable amphetamine-type stimulant named vint, prepared from over-the-counter medications including ephedrine (synonyms jeff, effect, boltushka; 4.2%); tramadol (prescription opiate analgesic, 3.8%); alcohol (1.9%); and marijuana (1.3%). Drugs used apart from opiates included vint (29.5%), ketamine (2.8%), and ecstasy (1.3%).

### HIV and HCV seroprevalence

HIV infection was detected in 44 participants (14.0%) and HCV infection in 230 (73.0%) out of the 315 total eligible IDUs. Thirty-eight participants (12.1%) were seropositive for both HIV and HCV; 192 (61.0%) had HCV only; and 6 (1.9%) had HIV only (Table 1).

**Table 1:** Prevalence of HIV and hepatitis C virus (HCV) infections among injection drug users in Vinnitsya, Ukraine.

HIV status # (%)	HCV status # (%)		
	
	Positive	Negative	Total
Positive	38 (12.1%)	6 (1.9%)	44 (14.0%)
Negative	192 (61.0%)	79 (25.1%)	271 (86.0%)
Total	230 (73.0%)	85 (27.0%)	315 (100.0%)

### Risky sex and drug use behaviors

About half of all participants (51.1%) reported having multiple sexual partners (>1) in the past year, and 10.5% exchanged sex for drugs or drugs for sex (see Table 2). Among all participants, 53.2% reported no or inconsistent use of condoms during sex. Almost half of the sample (41.3%) had injected drugs for more than 8 years; more than half (55.5%) used opiates regularly in the past year; and 74.6% used multiple drugs. Almost two-thirds (62.9%) reported sharing injection equipment in the past year while 8.0% knew they shared with an HIV+ person. Almost one-fourth (23.9%) back- or front-loaded a syringe one or more times per month in the past year. Almost half (44.8%) had received some form of treatment for drug addiction in their lifetime. More than half (53%) have ever used needle exchange programs. Participants also reported in qualitative interviews that operation of needle exchange programs was irregular, and many sites were undermined by police intercepting or approaching clients.

**Table 2:** Univariate analysis of categorical factors associated with HIV and hepatitis C virus (HCV) infections among injection drug users in Vinnitsya, Ukraine (Unadjusted odds ratios with 95% confidence interval).

Factors	No. of participants	No. of HIV positives (row %)	Unadjusted odds ratio (95% C.I.)	No. of HCV positives (row %)	Unadjusted odds ratio (95% C.I.)
**Sex**					
					
Female	57	8 (14.1)	1.0	41 (71.9)	1.0
Male	258	36 (14.0)	0.99 (0.5, 2.4)	189 (73.3)	1.01 (0.6, 2.0)
					
**Age**					
					
≤ 25 years	120	14 (11.7)	1.0	79 (65.8)	1.0
>25 years	195	30 (15.4)	1.4 (0.7, 2.8)	151 (77.4)	1.8 (1.1, 3.0) **
					
**Education**^§^					
					
High school or less	191	27 (14.1)	1.0	141 (73.8)	1.0
Some college or above	119	16 (13.5)	0.9 (0.5, 1.8)	85 (71.4)	0.9 (0.5, 1.5)
					
**Currently unemployed**					
					
No	99	16 (16.2)	1.0	72 (72.3)	1.0
Yes	216	28 (13.0)	0.8 (0.4, 1.5)	158 (73.2)	1.0 (0.6, 1.7)
					
**Number of sexual partners in the past year**					
					
≤1	154	23 (14.9)	1.0	114 (74.0)	1.0
>1	161	21 (13.0)	0.8 (0.4, 1.6)	116 (72.1)	0.9 (0.5, 1.5)
					
**Exchanging sex for drugs or drugs for sex in the past year**^§^					
					
No	278	40 (14.4)	1.0	207 (74.5)	1.0
Yes	33	3 (9.1)	0.6 (0.1, 1.8)	19 (57.6)	0.5 (0.2, 1.0) **
					
**No use or inconsistent use of condoms**					
					
No	145	22 (15.2)	1.0	106 (73.1)	1.0
Yes	165	21 (12.7)	0.8 (0.4, 1.6)	120 (72.7)	1.0 (0.6, 1.6)
					
**Years of using opiate drugs**					
					
≤ 8 years	185	27 (14.6)	1.0	124 (67.0)	1.0
>8 years	130	17 (13.1)	0.9 (0.5, 1.7)	106 (81.5)	2.2 (1.3, 3.8) ***
					
**Using opiate drugs daily or almost daily in the past year**					
					
No	137	12 (8.8)	1.0	90 (65.7)	1.0
Yes	171	32 (18.7)	2.4 (1.2, 4.9)*	137 (80.1)	2.1 (1.3, 3.5) ***
					
**Using multiple drugs in the past year**					
					
No	80	11 (13.8)	1.0	58 (72.5)	1.0
Yes	235	33 (14.0)	1.0 (0.5, 2.2)	172 (73.2)	1.0 (0.6, 1.8)
					
**Sharing needles or injection equipment in the past year**^§^					
					
No	116	11 (9.5)	1.0	80 (69.0)	1.0
Yes	197	32 (16.2)	1.9 (0.9, 4.0) *	148 (75.1)	1.4 (0.8, 2.3)
					
**Sharing needles with an HIV-positive person in the past year**^§^					
					
No	286	37 (12.9)	1.0	210 (73.4)	1.0
Yes	25	7 (28.0)	2.6(1.0, 6.5) **	17 (68.0)	0.8 (0.3, 1.9)
					
**Frequency of back- or front-loading in the past year**^§^					
					
Less than 1 time per month	236	33 (14.0)	1.0	162 (68.6)	1.0
One or more times per month	74	10 (13.5)	0.96 (0.5, 2.1)	63 (85.1)	2.6 (1.3, 5.3) **
					
**Ever received drug addiction treatment?**^§^					
					
No	171	19 (11.1)	1.0	110 (64.3)	1.0
Yes	139	24 (17.8)	1.7 (0.9, 3.2)	116 (83.5)	2.8 (1.6, 4.8) ***

**Note**.^§ ^There are missing data due to no response; * *P *< 0.1; ** *P *< 0.05; *** *P *< 0.01.

For the continuous risk variables (see Table 3), the average HIV-KQ-45 (HIV knowledge) score was 25.9 (SD = 7.6), which represents 57.6% of the items answered correctly (range 0 to100% correct) [17]. Average RAB drug risk score was 7.5 (SD = 4.4), falling in the high risk category (Norms: range 0–30, mean 5.04, SD 6.17, median 3). Average RAB sex risk score was 5.1 (SD = 2.0), also in the high risk category (Norms: range 0–27, mean 4.22, SD 3.5, median 4) [19]. The mean days of marijuana use in the past month (30 days) was 6.0 days (SD = 8.6).

**Table 3:** Univariate analysis of continuous factors associated with HIV and hepatitis C virus (HCV) infections among injection drug users in Vinnitsya, Ukraine (Mean ± SD; p-value for significance of mean differences using Student's t-test).

	Total	HIV-	HIV+	*p*-value	HCV-	HCV+	*p*-value
							
**HIV-KQ-45 score, points**							
							
	25.9 ± 7.6	25.3 ± 7.7	29.5 ± 6.4	0.0003	24.6 ± 7.7	26.4 ± 7.6	ns
							
**RAB drug risk score, points**							
							
	7.5 ± 4.4	7.5 ± 4.3	8.0 ± 5.0	Ns	6.7 ± 4.3	7.8 ± 4.5	0.04
							
**RAB sex risk score, points**							
							
	5.1 ± 2.0	5.1 ± 2.0	5.2 ± 2.3	Ns	5.2 ± 2.0	5.1 ± 2.0	ns
							
**Days of marijuana use in the past month**							
							
	6.0 ± 8.6	6.0 ± 8.6	5.9 ± 8.7	Ns	8.0 ± 10.7	5.3 ± 7.6	0.04

### HIV and HCV related risk factors

In univariate analyses, demographic, sex and drug-use risk variables were compared by HIV and HCV test results (Tables 2 and 3). Among demographic variables, only older age (>25 years) was associated with HCV seropositivity. Among the sex risk variables, past-year exchange of sex and drugs was negatively associated with HCV seropositivity. Among drug use risk variables, years of using opiate drugs (> 8 years) increased the risk of HCV. Recent and regular use of opiates doubled the risk of both HIV and HCV. Sharing needles or injection equipment and sharing with a known HIV positive person in the past year increased the risk of HIV 1.9 and 2.6 times, respectively. More frequent back- or front-loading of syringes and ever receiving drug addiction treatment increased the risk of HCV seropositivity by two-fold. Table 3 displays the univariate analyses of continuous variables associated with HIV and HCV infection. Greater HIV knowledge was associated with HIV seropositivity, and higher (worse) RAB drug risk scores and fewer days of marijuana use were associated with HCV seropositivity.

In multivariate analyses (Table 4), three variables were entered into the final model of predicting HIV infection, and all were retained. HIV seropositivity was positively associated with HIV knowledge (adjusted odds ratio (AOR), 1.1; 95% CI, 1.04, 1.15). Using opiate drugs daily or almost daily in the past year was associated with more than a two-fold increase in HIV risk (AOR, 2.2; 95% CI, 1.05–4.46). Those who had shared needles/syringes with an HIV positive person in the past year had more than three times the HIV risk of those who had not shared (AOR, 3.4; 95% CI, 1.24, 9.3). Seven variables were entered into the final model to predict HCV infection, and three were retained. Those who back- or front-loaded at least once per month in the past year had four times the HCV risk of those engaged in this practice less than one time per month (AOR, 4.0; 95% CI, 1.5–11.90). A one-day increase of marijuana use in the past month was associated with a 4% decrease of HCV risk (AOR, 0.96; 95% CI, 0.93–0.99). Those who had ever been treated for drug addiction or used a needle exchange had 2.4 times the risk of HCV compared to those who had never accessed these services (AOR, 2.4; 95% CI, 1.3–4.5).

**Table 4:** Multivariate logistic regression analysis of factors associated with HIV and hepatitis C virus (HCV) infections infection among injection drug users in Vinnitsya, Ukraine.

Variable	Model unit	Adjusted odds ratio	95% confidence interval	*p*-value
***Model: HIV (N = 268)***				
				
HIV knowledge (HIV-KQ-45) score	1 score point	1.1	1.04, 1.15	<0.001
				
Using opiate drugs daily or almost daily in the past year	Yes/no	2.2	1.05, 4.46	0.04
Sharing needles/syringes with HIV positive person in the past year	Yes/no	3.4	1.24, 9.3	0.02

				
***Model: HCV (N = 274)***				
				
Frequency of back- or frontloading in the past year	≥1 vs. <1 per month	4.0	1.5, 11.0	<0.01
Days of marijuana use in the past month	1 day of use	0.96	0.93, 0.99	0.02
				
Ever used drug addiction treatment	Yes/no	2.4	1.3, 4.5	<0.01

**Note**. Complete participant records from (N = 315) were excluded from the models if any variable was missing.

## Discussion

This study revealed that, in a community sample of injection drug users in central Ukraine (Vinnitsya Oblast), HIV and HCV prevalence are 14% and 73%, respectively, with co-infection among 12% of the IDU population sampled. These data provide evidence that HIV and HCV infections are prevalent among injection drug users of mostly hanka or home-made opiates in agricultural-based central Ukraine. The findings add to Booth's HIV prevalence findings of 34% in Kiev (major metropolitan area), 51% in Odessa (southern seaport), and 17% in Makeevka/Donesk (eastern mining region) in Ukraine [11]. They support the conclusion that multiple blood-borne infections among IDUs have reached epidemic proportions across Ukraine [20].

Central Ukraine seems to be experiencing one of the lower regional IDU HIV prevalence rates in this country. Differences in HIV prevalence by region may be explained by the history of the HIV epidemic. HIV was initially introduced in Kiev and southern seaport cities; spread from there to the east; and only later began to affect other regions [5,14]. By the time that larger scale testing and prevention programs were rolled out, the epidemic in Kiev and the south was in an advanced phase. Other regions were in an earlier (and thus lower prevalence) stage when voluntary counseling and testing and harm reduction programs became available. There are no reported differences in injection practices across different regions of Ukraine, although it is possible that more home-made hanka is used in Central Ukraine whereas more imported prepared heroin is available in larger cities and seaports. This, however, would seem to lead to greater HIV transmission among home-made hanka users due to more prevalent sharing practices. On the other hand, dependence on seasonal use (when the poppy plants can be harvested) may be associated with less use and ultimately less risk. The hypothesis that variations in either chemistry or in patterns of administration by IDUs may have a substantial role in shaping the epidemic was substantiated by Ciccarone and Bourgois among IDUs in the United States. They found that specific patterns of use (extra heating and cleaning of needles) of Mexican-derived heroin, commonly referred to as "black tar" heroin, may have retarded the spread of HIV in some U.S. states where black tar heroin predominates [21]. Further primary and secondary data sources and ethnographic research are needed to relate cultural IDU practices to HIV and HCV prevalence in Ukraine.

The cultural preparation, distribution and sharing practices of Central Ukraine are prime targets for prevention through education and, more importantly, through instituting accessible, stably operating needle exchange programs that can establish a first link between the IDU and the health care community. Furthermore, by attracting IDUs through street outreach and providing them with a source of clean needles and syringes, we then can advocate effectively for harm reduction, risk reduction, and HIV testing and provide prevention education and addiction treatment as demonstrated in other Ukrainian cities [8]. Our study confirms that the coverage of effective prevention programs such as needle exchange remains insufficient in the central region of Ukraine and should be scaled up to lower HIV incidence.

The phenomenon that HCV is more common than HIV among IDUs is well documented [22-25]. HIV and HCV share the major risk factor of percutaneous exposure, and small-volume percutaneous exposure is more effective for HCV transmission than for HIV transmission [26-28]. However, this study offers no clear scientific explanations as to why HCV is higher than HIV in these groups of injectors, given the findings that each infection was associated with unique but equally risky transmission injection practices: sharing needles with HIV-positive drug users (HIV+ group) and back- and front-loading (HCV+ group). Marijuana use was identified as a protective factor for HIV infection. Marijuana use may be associated with off-season periods when home-made hanka is harder and more expensive to get. Use of marijuana to relieve withdrawal symptoms and, ultimately, lack of hanka access may be associated with lower HIV risk (less frequent or more sporadic injection use).

Whereas HIV has been the focus of attention in harm reduction programs among IDUs in Ukraine and other European countries, HCV has been largely ignored and treatment perceived as too expensive. Our study findings underscore the importance of including diagnosis and basic care for HCV co-infection in HIV prevention and treatment programs. Antiretroviral therapy for HIV and interferon/ribavirin schemes for HCV have proven effective in reducing mortality and increasing quality of life [29-31]. These treatments are becoming more available in Ukraine, thus making early diagnosis vitally important for those infected. We found that 12% of study participants were co-infected with HIV and HCV. HCV may facilitate HIV disease progression and increase the incidence of liver toxicity associated with certain antiretroviral regimens. HCV co-infection represents a leading cause of morbidity and mortality among AIDS patients receiving antiretroviral therapy (ART) [32,33].

### Limitations

We used rapid screening tests for detecting HIV and HCV antibodies. We did not perform HCV RNA tests or recombinant immunoblot tests; therefore, false negative cases may exist in patients with severe cellular immune suppression as a result of HIV [34]. This may result in underestimation of HCV prevalence. HIV antibody screening may produce false positive results and lead to overestimation of HIV prevalence. However, the benefits of using rapid tests are increased participation and receipt of testing results and post-test counseling. The sample consists of volunteers recruited by a snowball sampling technique, beginning with seven "seeds." The strategy was quite effective in recruiting eligible IDUs from the home-made opiate injecting community, and a substantial proportion of the total estimated IDU population (380 out of estimated 2300 or 16.5%) participated. The sample does represent the networks of users initiated from the original 7 participants. But despite the fact that all initial participants were recruited from a narcological treatment facility, and therefore were included in the official registry of drug dependent persons at RND, our sample went beyond the treatment-exposed cluster of IDUs and included 55% who were not registered with the government treatment system. It appears that most participants were experienced users, as evidenced by high prevalence of physical stigmata of injection. Future studies should assure representation of new or careful injectors as well. Results of prior HCV and HIV testing were not used to exclude volunteers, which makes it difficult to identify true undiagnosed cases. Finally, there were no data collected indicating whether needle sharing preceded or followed HIV infection. To better differentiate HIV transmission versus acquisition risk behaviors, future research should strive to specify the circumstances surrounding HIV status, knowledge of status, and sharing behaviors.

## Conclusion

HIV and HCV are common among IDUs in central Ukraine. Efforts should be increased to implement education and harm reduction strategies to decrease unsanitary preparation and risky distribution and injection practices related to home-made opiate use in Central Ukraine. Governmental and non-governmental treatment of opiate IDU and HIV should also include testing and treatment of HCV. Harm reduction programs should be scaled up to reduce risks of HIV and HCV infection among IDUs and to prevent transmission to the general population through heterosexual contact.

## Competing interests

The authors declare that they have no competing interests.

## Authors' contributions

KD conceived of the study and participated in design, data collection, statistical analyses, and interpretation of results and led drafting of the manuscript. JS participated in study design and interpretation of results and helped to draft the manuscript. RS participated in study design and data collection. HQ participated in statistical analyses and interpretation of results and helped to draft the manuscript. OZ participated in data collection, statistical analyses and interpretation of results. SC participated in the literature review and interpretation of results and helped draft the manuscript. PS and LZ participated in study design, study implementation, data collection process, and interpretation of results. All authors read and approved the final manuscript.
